# Polysaccharides from Medicinal Plants: Bridging Ancestral Knowledge with Contemporary Science

**DOI:** 10.3390/plants13131721

**Published:** 2024-06-21

**Authors:** Lucas de Freitas Pedrosa, João Paulo Fabi

**Affiliations:** 1 Department of Food Science and Experimental Nutrition, School of Pharmaceutical Sciences, University of São Paulo, São Paulo 05508-000, SP, Brazil; lfpedrosa@usp.br; 2 Immunoendocrinology, Department of Pathology and Medical Biology, University Medical Center Groningen, University of Groningen, 9713 GZ Groningen, The Netherlands; 3 Food and Nutrition Research Center (NAPAN), University of São Paulo, São Paulo 05508-000, SP, Brazil; 4 Food Research Center (FoRC), CEPID-FAPESP (Research, Innovation and Dissemination Centers), Sâo Paulo 05508-080, SP, Brazil; 5 Food Research Center (FoRC), CEPIX-USP, University of São Paulo, São Paulo 05508-000, SP, Brazil

**Keywords:** medicinal, plant, polysaccharide, structure, function

## Abstract

Plants are a core part of cultural identity, as part of a diet, decorations, ceremonies, or as medicinal agents. Empirical knowledge regarding plants and their healing potential has existed worldwide for centuries. With the advance of science and technology, not only is the refinement of such sources or isolation of specific compounds possible, but these compounds can also be characterized based on their natural occurrence. Besides their importance for plant metabolism and structure, polysaccharides have been demonstrated to have substantial positive human health impacts on inflammation, metabolism, oxidative stress, and others. As an inherent part of plant cell walls, many polysaccharides from medicinal herbs, such as fructans, glucans, and pectins, have been extracted and analyzed for their structure and function. However, a review summarizing a significant portion of these studies was still unavailable. This review helps to fill the knowledge gap between polysaccharide bioactivity, their structure, and their plant matrix sources, focusing on historical medicinal usage.

## 1. Introduction

Traditional medicine is often referred to as the stem of modern medicine without its refinements. The WHO Global Report on Traditional and Complementary Medicine of 2019 defines it as the “total of the knowledge, skill, and practices based on the theories, beliefs, and Indigenous experiences of different cultures.” Of course, these are not always explained scientifically but have been used to diagnose and treat distinct human health and disease conditions throughout centuries [1,2,3].

One of the main branches is focused on the use of herbal medicines [4]. The components of regional plants, such as leaves, flowers, roots, and others, were and are still used, for example in teas, decoctions and infusions, to treat some diseases or symptoms, especially in more culturally conserved countries. Medicinal plants can be, therefore, defined as traditional or regional crops that somehow have phytochemical agents that can exert bioactive effects on health [5].

Advances in chemistry and pharmacology have promoted knowledge of the identification, isolation, characterization, purification, and even synthetical monetization of certain herbal components from medicinal plants. However, many other herbs that are of scientific interest still demand description and further studies. Techniques and extracts are constantly being studied for different plant species, but each plant and each type of extract, such as lipophilic oils, peptides, polysaccharides, or antioxidants, all have particularities of chemical extraction processes, which brings complexity to the matter [6,7,8,9,10].

It is noteworthy that plant polysaccharides are trending studied molecules for bioactive purposes. Polysaccharides are macromolecules composed of glycosidic subunits called monosaccharides. Both homopolymer and heteropolymer chains are common in nature. Those can be digested and absorbed by humans, such as conventional starch. Others cannot be digested by human enzymes and are defined as dietary fibers, such as pectins, β-glucans, resistant starch, and others. Structural complexity is another parameter that can differentiate between subclasses of polysaccharides. Besides elemental glycosidic composition, degree of branching, methylation, acetylation, linkage type, and conjugated structures contribute to molecular complexity [11,12].

Polysaccharides from medicinal plants are highly variable in their compositions. Fucose (Fuc), mannose (Man), galactose (Gal), glucose (Glc), and glucuronic acid (GlcA) are some of the sugars and uronic acids that can be present in different proportions and with distinct linkage types in those macromolecules. Fructans, α-glucans, and pectin-like polysaccharides are often isolated. Polyphenol conjugates and phosphor groups are also commonly found interacting with polysaccharides’ main or side chains [13,14]. In plant metabolism, polysaccharides are synthesized to give cell wall rigidity and stability, such is the case of pectins, for example, but can also perform as energetic reservoirs, being used similarly as glycogen in humans, such is the case of inulin, where it can be used to boost seedling growth and development.

Due to the importance of this topic, increasing interest in the literature, and the complex variety of extraction methods and polysaccharides resulting from them, this present review aims to give a descriptive overview of different extraction methodologies, plant sources, and biological outcomes of their medicinal applications. Besides the anthropological value, this can lead to a better overview of potential “target” plants that can be further explored.

## 2. Bibliometric Analysis

**Figure 1:**
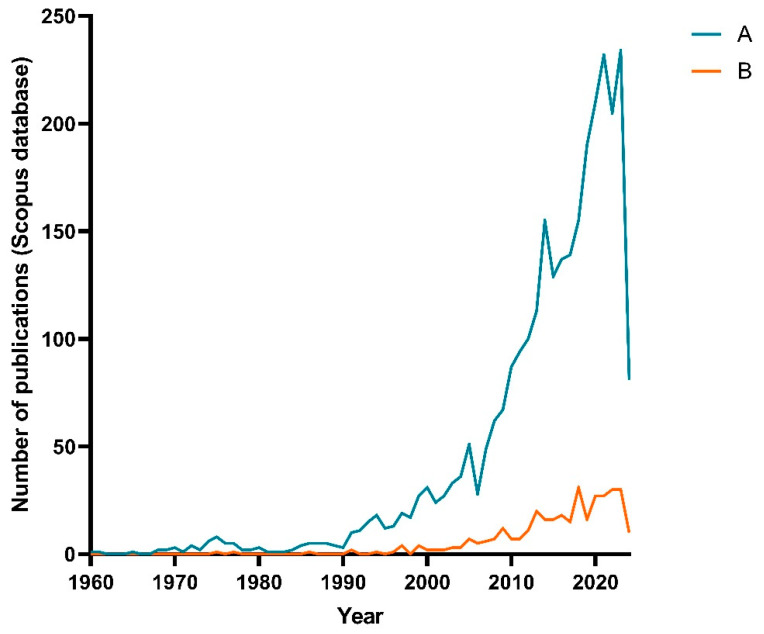
Number of publications over the years utilizing different descriptors. (A) Search containing terms “Polysaccharides”, “Extraction”, and “Plants”. (B) Search containing terms “Polysaccharides”, “Extraction”, “Medicinal” and “Plants”. For both search queries, “AND NOT” Boolean operator was used alongside terms “fungi”, “algae”, and “mushroom” to try to avoid misinterpretation of the number of articles found, since those terms are commonly associated with polysaccharides.

A bibliometric analysis was performed utilizing the Scopus database to provide scientific depth and perspective on applying medicinal plant-derived polysaccharides. Two independent searches were conducted (A and B) using the Boolean operators “AND” for three different terms: (A-1) “polysaccharides”; (A-2) “extraction”; (A-3) “plants”. For the second search (B), another term was included: “medicinal”. For both of them, “AND NOT” operator was used for three other different terms: (A-I) fungi; (A-II) algae; (A-III) mushrooms. This was needed further to specify the literature on the topic of interest. Then, the number of publications over the years was plotted as shown in Figure 1.

As expected, adding one additional term resulted in a more niche pool of articles found (131 documents indexed for B, against 852 for A, Figure 1). It is important to highlight that, even if there was no mention of the term “medicinal” in abstracts, keywords, or titles, the works found might still have been regarding some herb that would fit this category. Nevertheless, as a base of comparison, both search queries follow a similar trend of rising interest, especially starting around the 2000s.

**Figure 2:**
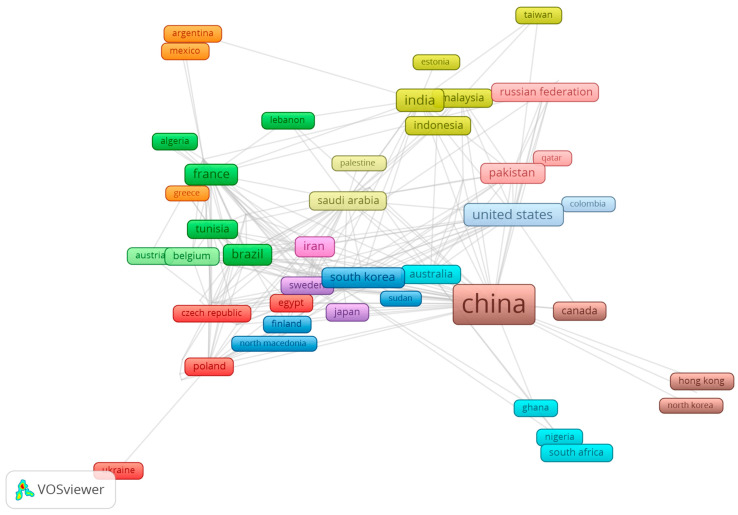
Cluster overview regarding country collaboration under the topic of the search query. The search was performed using the terms “Polysaccharides,” “Extraction,” “Medicinal,” and “Plants.” For both search queries, the “AND NOT” Boolean operator was used alongside the terms “fungi,” “algae,” and “mushroom” to try to avoid misinterpretation of the number of articles found since those terms are commonly associated with polysaccharides.

Next, networks of collaboration on publications regarding contributing countries were analyzed. A total of thirteen clusters were identified. Its main contributors, the number of countries inside each group, and the leading country in publications are detailed in Table 1, and visualized in Figure 2. The top three contributors worldwide were China, the United States, and India. The former had 8.7-fold more publications than the second contributor. Although China is the world’s leading scientific publication in most knowledge areas, ancient history might also play a role due to its population size and incentives for research production on this particular topic. One of the most famous and studied traditional medicine practices is Chinese (TCM), which rose in prominence between the 1960s and ‘70s, especially in Western societies, due to better translations of ancient TCM-related texts.

**Figure 3:**
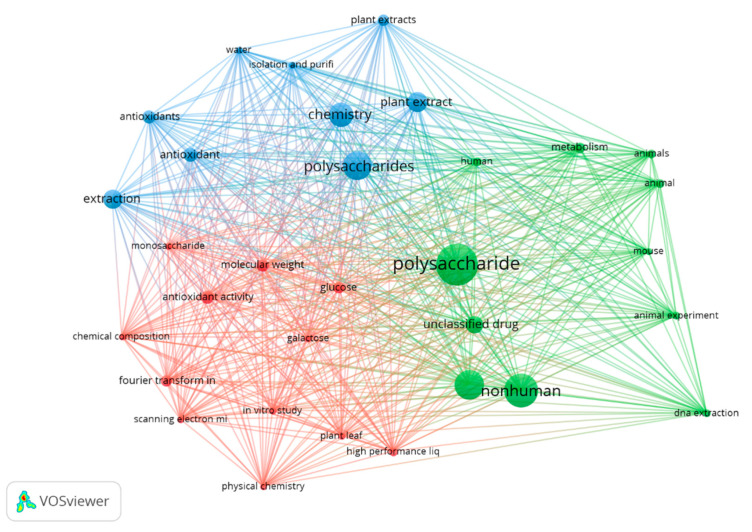
Cluster overview of keyword co-occurrence pattern from the above-mentioned search query. Search was performed containing terms “Polysaccharides”, “Extraction”, “Medicinal” and “Plants”. For both search queries, “AND NOT” Boolean operator was used alongside terms “fungi”, “algae”, and “mushroom” to try to avoid misinterpretation of the number of articles found, since those terms are commonly associated with polysaccharides.

Finally, the indexed keywords obtained from the broader research query were filtered for relevance, presented in Figure 3. Only keywords containing 100 or more co-occurrences are shown. This filter resulted in 32 keywords, divided into three major clusters.

Three major areas can be established for the observed clusters. (1) Function and form (Blue), which indicates the form in which the polysaccharides were tested or extracted. Terms such as “water”, “isolation and purification”, and “plant extracts” indicate typical procedures of the papers approaching medicinal plant polysaccharides. (2) Chemical characterization (Red): Common techniques used in polysaccharide analysis manuscripts for evaluation of structure, such as “Fourier transform infrared”, “scanning electron microscopy” and “high-performance liquid chromatography”, as well as the results obtained from such techniques, such as “molecular weight”, “monosaccharides”, and “chemical composition”. (3) Models of study (Green): Either the species where such molecules were tested (“human”, “mouse”, and “animal”) or the type of approach (“metabolism” and “DNA extraction”, for example).

## 3. Types of Polysaccharides

**Figure 4:**
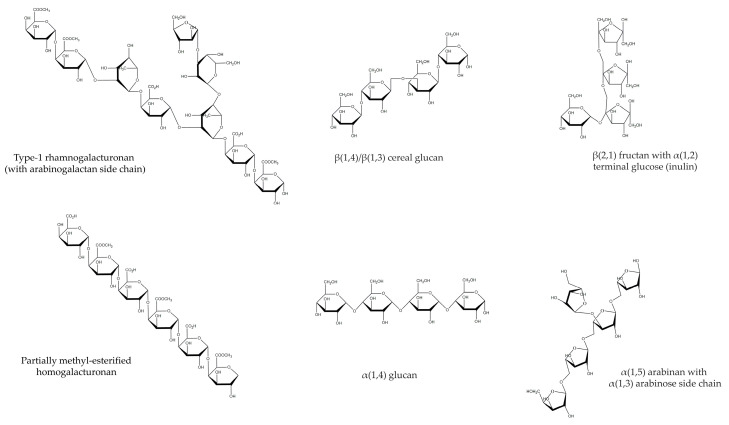
Chemical structure overview of the different polysaccharides discussed in the present review.

While medicinal plant extracts often contain a mixture of polysaccharides that can be conjugated with other bioactive and functional components, it is reasonable to summarize the most common polysaccharides found in plant materials (Figure 4). For this reason, the following topics will explore different types (e.g., fructans, hemicelluloses, and pectins), but also their effects on processes such as human inflammation, fibrosis, and metabolism.

### 3.1. Fructans

With the most commonly known representatives of this group being inulin, graminan, agavin, and levans, fructans consist of water-soluble oligo- or polymers with a fructose (Fru*f*) backbone, mainly thought to work as energy repositories in plants (not necessarily as structural components). Fructoses are bounded by β(2,1) and/or β(2,6) linkages [15,16]. It is also commonly described as having terminal glucosyl (Glc) residue. Inulin, in particular, is the most commonly known fructan with commercial value. It has been described as a prebiotic with great potential and interesting results related to health. Inulins are linear β(2,1) D-fructofuranose polymers, with terminal D-glucopyranose connected by α(1,2) glycosidic linkages. Their degree of polymerization—how many repeating Fru*f* residues are in each linear chain—is highly variable and dependable on sources. Elecampane, chicory (especially root), and Jerusalem artichokes are the most common industrial sources for inulin extraction [17]. Nevertheless, medicinal plants such as *Taraxacum officinale* L. (Dandelion) roots have been described as rich sources of this polysaccharide [18]. Inulins and other fructans have even been described as potential immunomodulators, mainly by interaction with pattern recognition receptors (PRRs) [19,20].

### 3.2. β-Glucans and Hemicelluloses

Beta-glucans are found in different biological domains, such as fungi (mushrooms and yeasts), bacteria, and plants. In plants, they are found in cereals such as oats, sorghum, wheat, and barley. Abundance is highly dependent on cultivar, but values from 1 to 20% (w/w%) have been described [21]. With intercalating sequences of DP2-4 β(1,4)-Glc*p* and one β(1,3)-Glc*p* residue, cereal β-glucans are homopolymers. β-glucans exert different beneficial biological effects, such as immunomodulation, antimicrobial profile, and anti-cancer properties in vitro/in vivo. Specific fractions of *Scutellaria barbata* D. Don, a Chinese medicine herb, have had specific structure features identified related to β-glucans, alongside differences observed by Su et al. [22] in anti-hepatoma activity from extracts.

### 3.3. Pectins, Arabinans, Galactans, and Arabinogalactans

Structural polysaccharides located in the middle lamella of plant cell walls, pectins are heteropolysaccharides with varied complexity, size, and composition. The main linear domain (smooth region) is composed of homogalacturonans, consisting of α(1,4)-Galactopyranuronic acid (Gal*p*A) units. Those can be intercalated with rhamnopyranoside (Rha*p*) through α(1,2) linkages, forming what is defined as rhamnogalacturonan (RG). Usually described as side-chains of pectin molecules, arabinans, galactans, and arabinogalactans compose an important fraction of both industrial crops and medicinal plants’ bioactive polysaccharides. Arabinans are α(1,5)-Arabinofuranoside (Ara*f*) homo-oligomer/polymers. Galactans are β(1,4)-Galactopyranoside (Gal*p*) oligomers or polymers that have been depicted as the bioactive part responsible for interactions with the protein Galectin-3 through carbohydrate recognition domain (CRD) [23,24,25]. Gal*p* and Ara*f* residues can be bound to each other in arabinogalactans, where the Ara*f* residues are located in the *O*-3 position of Gal*p* residues, either as monomers or oligomers. Arabinogalactans can also be side-chains of galactans themselves, where a Gal*p* residue is located at the *O*-6 position of another Gal*p* unit [26].

## 4. Potential Effects on Health

### 4.1. Immunomodulation

The human immune system is responsible for responsiveness to antigens and maintenance of homeostasis, reacting to the environment at variable degrees. A very energy-demanding tissue, this system comprises several organs and glands, with a complex mechanism of cell differentiation depending on activating and suppressive pathways [27,28]. The innate immune response is mainly operated through recognition and quick responses to specific molecules, such as pathogen- or damage-associated molecular patterns (PAMPs and DAMPs). One of the most antigen-exposed systems is the gastrointestinal system. The intestine has an intertwined specific immunological tissue called gut-associated lymphoid tissue (GALT) [29]. Both the epithelial gut and GALT cells are daily exposed to food components, bacterial antigens (mostly from intestinal microbiota), and other contaminants. Especially regarding the microbiota, different species also have distinct impacts on the intestinal threshold of tolerance and can be classified as pathogens or beneficial bacteria. Disruptions of the microbiota, named dysbiosis, are also responsible for disturbing the tolerance of human immune responses to specific strains, disrupting epithelial cell integrity and the mucus layer [30,31].

Many different polysaccharides from natural sources have been described as pro- or anti-inflammatory structures. This potential is probably due to molecular similarities to known/specific ligands of certain pattern recognition receptors (PRRs), for example, the glycan domain from lipopolysaccharides (LPS) or proteoglycans. In a cyclophosphamide (CTX)-induced immunosuppressed mouse model, *Caragana sinica* polysaccharides (CSP) were evaluated as potential immune restoration conditions. These polysaccharides are a mixture of arabinogalactan and glucans (monosaccharide ratio of 20:54:52, Ara:Gal:Glc). Levamisole and CSP-treated mice had boosted T and B cell lymphocyte proliferation compared to the control group. Those two groups also observed natural-killer (NK) and macrophage activation [32]. Cytokines are small signaling molecules related to immune responses. IL-1b, IL-6, IL-10, and TNF-α levels were also higher in the serum of those mice, indicating a restored response ability. In this case, the CSP had a pro-inflammatory profile, helping with potential infections in mice that were otherwise non-responsive, with pharmacological activity taking part in regulating immune function that may be related to the TLR4/MyD88/NF-κB pathway.

Another heteropolysaccharide composed of arabinose, galactose, and glucose, but this time from Chinese yam (*Dioscorea opposita Thunb* DOP), had its structure speculated as a main galactan chain, with linear β(1,3) and branched β(1,6) intervals, alongside α(1,4) Glc*p* side-chains. Again, phagocytic activity and proliferation of macrophages were higher in the polysaccharide treated in vitro. The polysaccharides boosted the immune system by increasing factors like NO, IL-6, and TNF-α, which helped immune cells communicate better. Also, in their mouse model, DOP reduced the damage caused by CTX in the immunosuppressed animals. Tissue disorganization was also attenuated in DOP-treated animals, especially regarding Thymus, spleen, and colon histopathology [33]. These polysaccharides promote the production of short-chain fatty acids (SCFAs) that are crucial in supporting gut health and immune function. By modulating the composition of gut microbiota, *Dioscotea opposita Thunb.* polysaccharides helped maintain the healthy balance of beneficial bacteria in the gut, remarked by changes in microbiota abundance, such as reduced Firmicutes/Bacteroidetes and a lower number of proteolytic/harmful bacteria. According to those results, the effects of polysaccharides are multifactorial, and different mechanisms might play a role in the observed effects.

*Glehnia littoralis*, an edible Asian medicinal plant, is rich in polysaccharides (GLP). Liu and colleagues (2024) analyzed the structure and bioactivity of GLP. Defined as branched α(1,5) and α(1,3–5) arabinan of about 7.7 kDa, GLPs exerted antitumor effect and inhibition of angiogenesis on a zebrafish model while promoting immune stimulation in vitro [34]. Molecular docking and surface plasmon resonance (SPR) assays were performed to evaluate the feasibility of interactions with TLR-4, PD-1, and VEGF proteins. TLR-4 stimulation would generate an inflammatory response, while PD-1 interaction could potentially inhibit PD-L1 binding and promote T-cell reactivation. VEGF is related to angiogenesis and is often a target for pharmacotherapy.

Another edible plant, especially its roots, *Lactuca sativa*, had water-extracted polysaccharides of alternating linear α(1,4) glucan with *O*-6 Glc*p* terminal residue, often in the chain’s branches. The authors identified a pro-inflammatory profile, where phagocytosis rate, NO concentration, and IL-1b, IL-6, and TNF-a levels were increased after treatment in a dose-response manner [35]. *Pueraria lobata*, a known herbal medicine with α-glucan polysaccharides, induced IL-2, IL-4, and TNF-a increase, enhancing specific T and B cell populations in vitro [36]. This backbone of α(1,4) glucans has been described as having pro-inflammatory properties in sources other than plants, such as clams and fungi [37,38]. The polysaccharides obtained from the *Arctium* genus after fermentation by *Rhizopus nigricans* (a common brown-bread fungus) were isolated and tested in in vitro and CTX models. TLR-4, MAPK, and NF-kB pathways were activated by those polysaccharides, and besides the already described above common effects, tight-junction proteins such as intestinal claudin-5 and occluding expressions were upregulated in the mice [39]. Those tight-junction proteins help to maintain epithelial cell-to-cell integrity, lowering the exacerbated exposure of the intestinal lamina propria to undesired pathogens.

Two polysaccharides from *Portulaca oleracea* L., one of the widest-used medicinal herbs, were extracted and fractionated through anionic exchange columns. While POL-1 was a linear low-methyl esterified homogalacturonan (α-1,4-Gal*p*A), POL-2 consisted of a linear galactan chain (β-1,4-Gal*p*) with glucan branches (α-1,4-Glc*p*). Size was also different between both (64 × 21 kDa, respectively). The authors observed and described a rise in macrophage phagocytic activity, NO secretion, and cytokine production (TNF-a and IL-6), but only for POL-2. POL-1. On the other hand, it did not present any immunostimulatory activity [40]. Interestingly, this trend was exactly the opposite when *Crataegus* spp. (Hawthorn) pectic polysaccharides were analyzed, where the fraction with more Gal*p*A was responsible for higher NO release and phagocytic activity. This immune stimulatory profile was concluded to be via TLR4 binding, with MyD88 and TRAF6 upregulation in the macrophages [41]. Once again, this highlights the variability between medicinal plant species polysaccharides, even if they have similar major components. Other structural aspects, either by natural occurrence or extraction methods, are responsible for different biological effects.

### 4.2. Fibrosis and Hepatoprotection

The liver is the detoxification center of the human body. It holds a balance between metabolizing components retrieved exo- and endogenously. During imbalance, either by overcharge or damage, the net product of oxidant products might be higher than expected, inducing higher oxidative pressure toward hepatocytes. One of the sources of liver damage is autoimmune hepatitis (AIH). Autoantibodies for liver proteins/cells are generated, and immune cell infiltration is higher than baseline. This often leads to fibrosis and cirrhosis. ALT and AST transaminases are two biomarkers for liver damage. A very purple sweet potato (*Dioscorea alata*) polysaccharide (PSPP) consisting of high glucose, followed by galacturonic acid and galactose, lowered ALT and AST in Con A liver-injury-induced mice. The polysaccharide also blocked hepatocyte apoptosis through Bax and Bcl-2 expression at its higher dose, lowering oxidative stress-related markers [42].

Hepatic stellate cells (HSCs) are a minor composition of liver tissue that responds to a stress-secreting collagen-rich matrix. Its overactivation leads to liver fibrosis. A series of inulin-like fructans performed well against different models of fibrosis. *Achyranthes bidendata* is a Chinese herbal medicine with properties related to kidney and liver health. Carbon tetrachloride (CCl_4_) is a toxic chemical to the liver, inducing fibrosis and acute hepatocyte stress in mice. Inulin-like fructans from this herb were tested in a CCl_4_-induced mouse model, improving liver fibrosis and inhibiting HSC activation after intraperitoneal administration. AST and ALT plasma levels were also decreased at three and seven weeks of treatment, compared to the positive control, denoting less liver stress over the course of the experiment. The polysaccharide inhibited the signaling of the FAK/PI3K/AKT pathway in vitro [43]. *Ophiopogonis Radix* fructans helped regulate collagen deposition, such as lower α-SMA and collagen, while having anti-inflammatory and anti-apoptotic properties on liver tissue [44]. Xiebai, or *Allium macrostemon* and *Allium chinense* G. Don polysaccharides (AMB and ACGD), renowned as a food and herbal medicine in China, reduced myocardial fibrosis in vivo compared to the model Apoe-/- high-fat and cholesterol diet animals without the polysaccharide administration. Their molecular structure was also an inulin-like polysaccharide, with β(2,6) Fruf residues and an α(1,6) Glc*p* terminal [45].

*Arctium lappa* L. is called Niubang in China, and the common name in English is Burdock. Over the centuries, it has been a synonym for health-promoting and traditional medicine herbs. Treating fever, dizziness, sore throats, infection and others are some of the described effects, without focusing on certain isolated products from the plant [46]. Lu et al. [47], however, isolated heteropolysaccharides composed of glucose, arabinose, and galactose from the roots of *Arctium lappa* (ALP), and observed anti-inflammatory effects in lung models. Later, Xiang et al. [48] tested the same extracted polysaccharides in an in vitro model of nasal ectomesenchymal stem cells (EMSCs) to prevent liver fibrosis by inhibiting the Wnt/β-Catenin pathway. EMSCs were exposed to the polysaccharides, and parameters such as antioxidant activity were measured. ALP-treated EMSCs did not lose stemness while enhancing their anti-inflammatory and paracrine activity. Non-conditioned EMSCs were already able to reduce the fibrotic activity of HSCs alone, but when submitted to the ALP pre-condition, the effects were more pronounced [48].

### 4.3. Diabetes, Lipid Metabolism, and Glucose Tolerance

Diabetes is a pathology where the patient is constantly under hyperglycemia. Most commonly defined between type 1 and 2 diabetes, emerging classifications such as latent autoimmune diabetes of adults (LADA) and other pathogenesis models are described [49]. The former has a marked auto-immune role. The immune system detects β-pancreatic cells as potential threats, decreasing β-pancreatic cells and lowering insulin production and secretion capability. Type 2 diabetes is a very complex metabolic disease, characterized as being heterogeneous, that is, also having an immune background, but with the environment playing a great role in its onset. Dietary patterns, exercise, exposure to contaminants, and others are some factors associated with pathogenesis progression [50]. While the role of dietary fiber is already quite well known regarding cholesterol-reducing potential, fasting insulin and glucose regulation, glycated hemoglobin (HbA1c) and other insulin-related parameters (such as HOMA-IR) [51], specific mechanisms of polysaccharides derived from medicinal plants have also been identified.

*Amorphophallus konjac* is a native Southeast Asian plant, rich in glucomannan polysaccharides (KGM). Different hydration profiles and delivery methods have been tested in healthy adults, and medium hydration and viscosity (MHMV) glucomannans had the best postprandial glycemia of all groups. Meanwhile, faster hydration and higher viscosity (FHHV) had the best appetite-controlling ability [52]. KGM were also administered alongside *Polygonatum cyrtonema* Hua polysaccharides (PCP) in mice. PCP consisted of a long chain fructan (240 kDa). KGM and PCP supplementation helped attenuate the body weight and specific tissue loss of the animals submitted to streptozotocin (STZ) injection. Furthermore, the combination of both reduced HOMA-IR, increased HDL cholesterol, and decreased LDL-c and triglycerides. Hepatocellular lesions were also controlled. Both physicochemical characteristics, such as the viscosity and microbiota composition modulation, were described as potentially playing a beneficial role [53].

*Cyclocarya paliurus* (CP) is a traditional Chinese medicine herb, of which mainly the leaves are used. CP polysaccharides (CPP) are one of the main constituents of plant leaves. Although the specific structures of the polysaccharides were not determined, certain fractions could inhibit α-glucosidase activity, protect β-TC-6 cells from apoptosis, and restore glucose-analog uptake by HepG2 cells [54]. Another species composing the Chinese pharmacopeia is *Glycyrrhiza uralensis* (*GU*). Its polysaccharide extract was found to have a purity of 90% carbohydrate content, 230 kDa average molecular weight, and was mostly composed of Man*p* and Gal*p* residues [55,56]. In an STZ-induced mouse model, GU-Polysaccharides (GUP) were able to restore glucose homeostasis of the diabetic animals while also lowering lipid levels and liver compromise. At a microbiome level, it lowered the overall abundance of Firmicutes and raised Bacteroidetes, a profile attributed to insulin resistance in another previous study [56,57].

**Figure 5:**
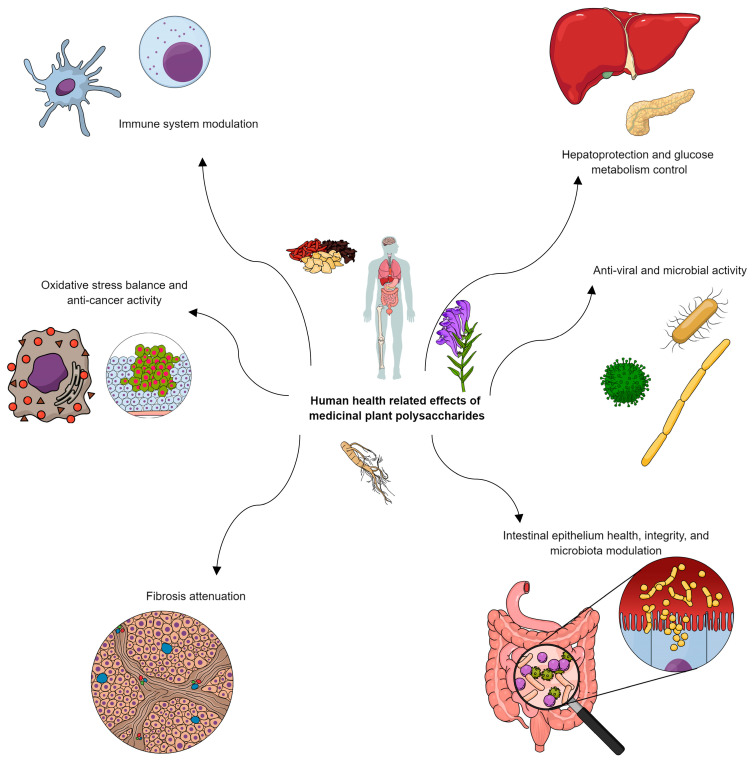
Summarized biological effects from medicinal plant polysaccharides on human health.

Diabetes is known to disturb lipid metabolism. One of the pathways involving lipid homeostasis is through sterol regulatory element-binding protein (SREBP). Mice were fed a high-fat diet to generate insulin resistance and were induced with STZ to be diabetic. A heteropolysaccharide from *Arctium lappa* L. (ALP) composed mainly of fructose, galactose, and xylose reverted liver weight gain while controlling animal weight gain throughout the experiment [58]. Total cholesterol, LDL-c, HDL-C, and TG were lower in the ALP-treated group. By immunohistochemistry and Western blotting, the overexpression of both SREBP and its downstream protein, SCD-1, was detected in the diabetes model group livers, while not on ALP-treated diabetic mice [58]. This reinforces the physicochemical aspect of glucose tolerance and homeostasis and further systemic responses towards certain polysaccharides, if solely by microbiota modulation or other signal transducing mechanisms. Direct inhibition effects on carbohydrate hydrolases, such as α-glucosidase and α-amylase, were also observed for some polysaccharides, such as the heteropolysaccharides from *Scutellaria baicalensis*, composed mainly of two uronic acids, glucuronic and galacturonic, and one neutral sugar, galactose [59]. Below is a summary of some of the already discussed medicinal plants, their extraction methods, described polysaccharide structures, the model on which they were tested, and biological effects. Other non-discussed sources are also in the table (Table 2). The discussed biological effects are also summarized in Figure 5.

## 5. Other Functional Groups Conjugated to Polysaccharides

Although having a complex structure with varied bioactivities, natural and synthetic modifications can be performed on polysaccharide backbones such as phosphorylation, amination, selenization, and others. A great review focusing solely on the antioxidant profile of polysaccharides, with special observations regarding substituted groups and conjugates, has been made before [91]. The focus here will be once more specifically on medicinal plants’ polysaccharides and what information we have until now regarding reports of those, focusing whenever possible on health effects.

### 5.1. Phosphorylated Polysaccharides

*Ginkgo biloba* L. is a very traditional herb utilized as medicine, and it has a vast history, mainly in China. The leaves and fruits are rich in many compounds, such as polysaccharides, phenolics, and terpenoids [92]. From the leaves, Li et al. [93] extracted and purified a heteropolysaccharide of α-Rha, β-Gal, α-GlcA, and β-Man. Then, they chemically phosphorylated the obtained polysaccharide through a phosphoryl chloride–pyridine method, retrieving slightly more than 10% of phosphorylation. While both polysaccharides exerted anti-oxidant properties for hydroxyl radicals, lipid peroxidation, and DPPH assay, the phosphorylated version performed much better [93] in an experiment that represents only the use of polysaccharides to produce other bioactive molecules.

CP polysaccharides were also phosphorylated, this time through a sodium tripolyphosphate and sodium trimetaphosphate method. Besides having more DPPH and hydroxyl scavenging rate than its natural correspondent, phosphorylated CP (P-CP) ameliorated oxidative stress-related markers in RAW 264.7 cells. Hydrogen peroxide (H_2_O_2_) is a common method to evaluate cellular oxidative damage. CP and P-CP did not alter cell viability, but were able to recoup part of the decrease from H_2_O_2_ exposure, lowering reactive oxygen species (ROS) and malonaldehyde, and enhancing superoxide dismutase (SOD) enzyme activity [94].

*Momordica charantia* L., or bitter melon, is an edible and medicinal plant from the Cucubitaceae family. Despite unclear information on polysaccharide structure, Chen et al. [95] described a phosphorylated derivative that improved mice serum, liver, and spleen markers for oxidative stress. Malonaldehyde contents were dose-dependently lower, while catalase (CAT) and SOD contents were higher. However, specific assays of activity or expression of downstream pathways were not performed [95]. As mentioned briefly by the authors and reinforced here, despite the positive effects observed on those phosphorylated polysaccharide derivatives, specific structure–function correlations, further experimentation with more refined structural characterization, and finally, more in vivo models to evaluate different plausible mechanisms of action are needed to improve this discussion.

### 5.2. Polyphenols and Organic Acids

Polyphenols and polysaccharides can naturally interact through hydrophobic, hydrogen-bound, non-covalent ion, and covalent interactions. Synergistic beneficial health effects might exist when those molecules are conjugated. For example, Mulberry leaf (*Morus alba* L.) polysaccharides and polyphenols were extracted and then combined in a ratio of 2:1 (PPPS). Their combination enhanced polyphenol digestion in vitro, while monosaccharide release remained unchanged. Accordingly, after simulated colon fermentation, the short-chain fatty acid (SCFA) production pattern was changed, especially regarding propionic acid after 12 h of fermentation [96]. Such changes were also accompanied by microbiota modulation, and the authors suggest that the shift in microbial profile and diversity could be one of the reasons for previously identified biological outcomes in vitro and in vivo [96,97,98]. Similar bioavailability results were also found for polyphenols bound to *Lycium barbarum* L. traditional medicinal plant, where the digestion dynamics for the conjugates were more complex than polyphenols alone. Nevertheless, more bound phenolics were released after digestion, and microbiota diversity differed from the control group [99].

Olennikov et al. [83] extracted and purified different fractions of polysaccharide fractions from *Vaccinium vitis-idaea* (Lingonberry) waste. After further purification of the most antioxidant fraction, the authors found and described two distinct classes of polymers, with two representatives in each: (1) polyphenol–polysaccharide conjugates, with a neutral arabinogalactan backbone (suggested as linear 1-3-Gal*p* with *O*-6 bound 1-3-Ara*f*) and hydroxycinnamoyl fragments (mainly sinapic and ferulic acids), and (2) mainly acidic pectin polysaccharides (1-4-Gal*p*A). After the conjugates performed much better in vitro on antioxidant potential for DPPH, ABTS, O_2_- and OH-, and had a better bile-acid-binding potential, they were administered to animals with standard and high-fat diets. While both polysaccharides were able to control animal body weight, TG, LDL-c, HDl-c, and total cholesterol levels, the polyphenol–polysaccharide conjugates were much more effective in controlling oxidative stress parameters, such as increasing liver antioxidant enzymes CAT, SOD, and glutathione peroxidase (GPX), and lowering serum MDA [83].

Two polyphenol–polysaccharides were extracted from *Tamarix chinensis* L. Both had a phenolic content between 12 and 15%; while one was composed mainly of a mixture of linear α and β(1,4)Glc*p*, with *O*-3 α(1,3)Ara*f*, and β(1,4)Gal*p*, the other was only composed of alternating α(1,3–4)Glc*p* and *O*-2 methyl glucosides. The most significant phenolic compound identified was *O*-6-bound quercetin. Through an in vitro hemolytic assay, the first conjugate and quercetin alone had comparable results to heparin, with great anti-complement activity [82]. Interestingly, this herb has been used for treating measles and pneumonia-complicated measles in traditional Chinese medicine. Again, the same scientific group used the crude polysaccharide (non-fractionated) and another polysaccharide but now substituted with myricetin. Those polysaccharides were then tested on an H1N1 acute lung injury mice model. The highest dose-administered animals (400 mg/kg) had a much higher average survival rate when compared to the model group, as well as improving body weight maintenance and alveolar destruction/lung tissue inflammation [81]. The authors also found higher levels of the anti-inflammatory cytokine IL-10, while lower quantities of MCP-1 and IL-8, macrophage chemokines. Another quercetin-substituted polysaccharide from the same crude polysaccharide was also tested in H1N1-infected mice, and doses of 50 and 100 mg/kg could attenuate lung injury and inflammation once more. Besides similar cytokine profile modulation, the authors found evidence of inflammasome (NLRP-3) and TLR4/NF-kB down-regulation properties [80]. This extensive report on data available regarding medicinal plant polysaccharide biological and chemical evaluation supports the already vastly employed empirical usage of such plants, but with a scientific approach and detailed observation, complementing the ancestral knowledge of this subject.

## 6. Conclusions

Plants are an inherent part of human eco-cultural interaction. They are part of ceremonies, dietary patterns, decorations, and medicinal use. Medicinal plants are those herbs that have been described throughout centuries of empirical trial and error in many different contexts by many distinct ethnic groups. As sources of varied compounds, such as terpenoids, phenolics, minerals, vitamins, essential oils, and polysaccharides, their structural analysis and fragmentation are fundamental to enhancing our scientific understanding of such precious matrixes. Polysaccharides can be significantly abundant parts of a plant structure, and their health-related effects have previously been reported in great numbers. This review was able to characterize the actual bibliographic perspective on medicinal plants and their polysaccharides, especially regarding their structure and related functions on in vitro and in vivo models, summarizing it. Besides polysaccharide main chains, other plant-metabolic products might be conjugated to it, also enhancing such effects, and a couple of recent examples were also discussed here. Overall, such review aimed to link human ancestral knowledge with the scientific method, as we believe such a link is a powerful tool for health, but also social sciences. We expect that such work can be of use for future research studying different plant species and their respective polysaccharide structures, strengthening the bridge between structure, effect, and usage, both in science and society.

## Figures and Tables

**Table 1:** Cluster number, color, principal contributors, number of countries, and top contributing country based on the bibliometric data analysis performed on VOS Viewer version 1.6.20 software.

Cluster	Cluster Color	Main Contributors	Number of Countries inside Cluster	Top Contributing Country (Documents)
1	Dark red	Poland, Egypt, Czech Republic	10	Poland (15)
2	Dark green	France, Brazil, Tunisia	10	France (42)
3	Dark blue	South Korea, Thailand, Vietnam	9	South Korea (26)
4	Golden yellow	India, Malaysia, Indonesia	8	India (54)
5	Purple	United Kingdom, Germany, Sweden	8	United Kingdom (23)
6	Turquoise	Australia, South Africa, United Arab Emirates	7	Australia (22)
7	Orange	Spain, Argentina, Chile	6	Spain (18)
8	Copper	China, Canada, Hong Kong	5	China (480)
9	Pink	Iran, Italy, Turkey	5	Iran (35)
10	Light red	Pakistan, Russian Federation, Bangladesh	5	Pakistan (25)
11	Light green	Belgium, Austria	4	Belgium (10)
12	Light blue	United States, Colombia, Ecuador	3	United States (55)
13	Light yellow	Saudi Arabia, Palestine	2	Saudi Arabia (24)

**Table 2:** Summary of medicinal plant names, extraction method, polysaccharide structure, model and biological effects observed in such models.

Plant	Extraction Method	Polysaccharide Structure	Model	Biologic Effects Observed	References
*Caragana sinica*	Hot water extraction	Arabinogalactan, 5 kDa	In vivo	Immunostimulatory potential of CTX immunosuppressed mice, through TLR4/MyD88/TRAF6	[32]
*Dioscotea opposite*	Hot water extraction with α-amylase	β-galactan backbone with α-glucan branches, 1500 kDa	In vivo	Immunostimulatory effects on CTX immunosuppressed mice, microbiota modulation	[33]
*Glehnia littoralis*	Hot water extraction	α-arabinan, 7 kDa	In vitro and in vivo	Antitumor activity on zebrafish, dendritic cell maturation, upregulation of cytokines and phagocytic activity	[34]
*Lactuca sativa*	Hot water extraction	α-glucan with O-6 α-Glc terminal residues, 22 kDa	In vitro	Improvement of proliferation and phagocytosis of macrophages, NO and cytokines upregulation	[35]
*Pueraria lobata*	Hot water extraction	α-glucan with α-Glcp branches, 14 kDa	In vitro and in vivo	Reversion of CTX induced immunosuppression, stimulation of T, B and macrophage cells activity and proliferation	[36]
*Sinonovacula constricta*	Hot water extraction and papain	linear α-1,4-glucan, 15 kDa	In vitro	Macrophage viability increase, phagocytic activity stimulation, NO/TNF-a/IFN-y/IL-1b promotion	[37]
*Portulaca oleracea* L.	Hot water extraction	Two polymers, 64 kDa 9% DM homogalacturonan; 21 kDa β-galactan with O-6 substituted α-glucans	In vitro	Immunostimulant of macrophage phagocytosis and cytokine release	[40]
*Crataegus* spp.	Hot water extraction	One parent and two fraction polymers; both fractions had similar Ara and Gal, but different Glc and GalA	In vitro	Higher macrophage phagocytic activity and NO release, as well as cytokine production via TLR4 downstream pathways, especially for the polymer with more GalA	[41]
*Dioscorea alata*	Hot water extraction	No size described, composed of Glc, GalA, Gal, Ara, Rha and GlcA, in this order of importance	In vivo	Reduced hepatic apoptosis, damage, elevation of AST and ALT, by suppressing TLR4-P2 × 7R/NLRP3	[42]
*Achyranthes bidentata* Bl.	Hot water extraction	β-Fructan with mainly DP4, but with DP from 2 to 12	In vitro and in vivo	Attenuated liver damage and fibrosis induced by CCl4 on mice, inhibited HSCs migration and proliferation	[43]
*Ophiopogonis radix*	Hot water extraction	β-fructan with 2-6-β-fructan side chains, 5.76 kDa	In vivo	Hepatoprotection, attenuation of collagen deposition, inflammation via MAPK and TGF-b/Smad pathways	[44]
*Allium macrostemon* Bunge *and Allium chinense* G. Don	Hot water extraction	Respectively, 25 and 19 kDa, both with major abundance of Fru, and minor amounts of Glc	In vitro and in vivo	Reduced hypoxia/reoxygenation induced apoptosis via Bcl2/Bax pathway, myocardial protection	[45]
*Arctium lappa* L.	Hot water extraction	Glucan, 2 kDa, with Gal and Ara as secondary monosaccharides	In vitro and in vivo	Antioxidant enzyme activity enhancement, anti-inflammatory profile; lipid metabolism regulation through SREBP/SCD-1 axis; anti-fibrotic action	[47,48,58]
*Amorphophallus konjac*	Hot water extraction	Glucomannan, >1000 kDa	In vivo	Slower glucose diffusion, lower post-prandial glucose levels	[52]
*Cyclocarya paliurus*	Ultrasonic and enzymatic	Mw varying from 24 to 100 kDa-, GalA-, Glc-, Gal- and Ara-containing polysaccharides and their phosphorylated derivatives	In vitro	Antibacterial activity, oxidative stress protection on IEC-6, enhanced insulin secretion in pancreatic β-cells	[54,60]
*Glycyrrhiza* spp.	Hot water extraction	Mixture of Xyl-, Man-, Glc- and Gal-containing polysaccharides	In vitro and in vivo	Antioxidant properties in vitro; in STZ-induced mice, lower nitrogen and creatinine levels in serum, intestinal barrier enhancing, hyperglycemia and insulin resistance attenuation, and others	[55,56]
*Moringa oleifera* Lam.	Hot water extraction with UV/H2O2 follow-up	Between 2 and 3 kDa branched arabinogalactans	In vitro human fermentation	Prebiotic effects: higher beneficial bacteria abundance and promotion of SCFA production	[61]
*Scutellaria barbata* D. Don	Hot water extraction	Two polymers, 110 and 140 kDa arabinogalactans with different abundances of Xyl and Glc residues	In vitro and in vivo	Hepatocellular carcinoma G1 phase arrest, apoptosis induction, through p53 and bax/bcl-2 upregulation	[62]
*Angelica sinensis*	Hot water extraction	Partially methyl-esterified galactan, 80 kDa, with Araf and Glcp branches, and terminal GlcpA	In vitro and in vivo	Citotoxicity to tumor cells; inhibited fibrogenesis in RLE-6TN cells, suppressed pulmonary fibrosis in mice, regulation of DANCR/AUF-1/FOXO3 axis	[63,64]
*Tetrastigma hemsleyanum*	Hot water extraction	Polymer composed of, in order of abundance, GalA, Glc, Man, Ara, Gal, and Rha, 66 kDa	In vitro and in vivo	Anti-tumor and anti-pyretic via TLR4/NF-kB, antibiotic-induced intestinal barrier dysfunction attenuation,	[65,66,67,68]
*Inula japonica*	Hot water extraction	α-Araf arabinan with variable glycosidic linkages (5-1/3,5-1/2,3,5-1/2,5-1), and lower amounts of Glcp and Galp, 100 kDa	In vitro and in vivo	Antitumor activity on zebrafish; inhibition of angiogenesis and immune activation via TLR4/PD-1/VEGF	[69]
*Dendrobium officinale*	Hot water extraction	Heterogenous glucomannan	In vivo	Attenuation of gastric ulcer through oxidative stress regulation, anti-apoptosis and inflammatory properties	[70]
*Onosma glomeratum* Y. L. Liu	Non-hot water extraction	Pectic polysaccharide, with linear homogalacturonan and RG-I domains with arabinogalactan side-chains, 62 kDa	In vitro and in vivo	Anti-inflammatory effects, diminishing effect of LPS-induced pulmonary inflammation through NF-kB pathway	[71]
*Daphne mezereum* L.	Hot water extraction	Two polymers, one neutral mixture of arabinan, arabinogalactan and hemicellulose, other acidic pectic polysaccharide (one neutral subfraction was also obtained)	In vitro	IFN-y and TNF-a secretion stimulation on PBMCs, especially for the neutral fractions	[72]
*Phoenix Dactylifera*	Hot and cold-water extraction	Arabinogalactan and xyloglucan mixture in one study; 246 kDa with GalA, Glc, Man, Fru, and Gal in another study	In vivo	Reduced toxicity from a single-cisplatin injection on rats identified through liver tissue and biomarkers, oxidative stress and cytokines; antimicrobial and antitumor activity	[73,74]
*Bletilla striata*	Hot water extraction	Glucomannan, 50 kDa	In vivo	Lung fibrosis attenuation in mice through inhibition of lung fibroblast activation, proliferation and migration, autophagy stimulation and TGF-B1/Smad pathway suppression; anti-inflammatory and analgesic	[75,76]
*Codonopsis pilosula*	Hot water extraction	β-1,2-Fructan with terminal α-Glc residues, 5 kDa	In vitro and In vivo	Increased activity of liver antioxidant enzymes, lower liver index, body weight, body fat index and increased liver function in NAFLD model mice	[77]
*Dolichos lablab* L.	Hot water extraction	Ara, Gal, Glc, and GalA polysaccharide with bound flavonoids and phenolics	In vivo	Microbiota modulation, anti-ulcerative and inflammatory properties	[78]
*Stemona tuberosa*	Hot water extraction	Six different polysaccharides with varying Mw from 10 to 250 kDa; all of them had Gal, while the root-derived ones had higher Glc and lower GalA contents; both stem polysaccharides had higher GalA	In vitro	Leaf polysaccharides inhibited MUC5AC overexpression, while root polysaccharides had anti-inflammatory properties	[79]
*Tamarix chinensis* L.	Hot water extraction	Four different phenolic-substituted polysaccharides with quercetin or myricetin, with α-glucan backbone and arabinan branches	In vitro and in vivo	Anti-oxidant, anti-complement, and anti-inflammatory properties, TLR4/NF-kB inhibition, on H1N1-induced mice	[80,81,82]
*Vaccinium vitis-idaea*	Hot water extraction	Two acidic polysaccharides and two neutral arabinogalactan–polyphenol conjugates varying from 108 to 318 kDa	In vitro and in vivo	Liver anti-oxidant enzyme activity enhancement, serum cholesterol- and triglyceride-lowering properties	[83]
*Scutellaria baicalensis*	Ultra-sound assisted and enzymatic extraction	Mainly composed of GlcA, GalA, Gal, and Glc, 89.7 kDa	In vitro	Dendritic cell activation, α-amylase and α-glucosidase inhibition	[59]
*Lilium lancifolium*	Hot water extraction	O-2-acetyl linear glucomannan, 5.3 kDa	In vitro	Anti-oxidative stress through Nrf2/HO-1 pathway on HUVEC cells	[84]
*Smilax china* L.	Enzymatic assisted hot water extraction	Heteropolysaccharides of Ara, Gal, Glc Xyl, and GalA, 134 kDa	In vivo	Reversion of high-fat diet body weight, liver, and adipose tissue weight, upregulation of lipidolysis genes and proteins	[85]
*Morus alba* L.	Hot water extraction	Heterogenous polysaccharide with 200–800 kDa mainly composed of Glc, followed by GalA, Gal and Ara	In vivo	Microbiota modulation, reduced adipose tissue, improved insulin resistance, and improved pathological lesion in the colon	[86]
*Rosa chinensis*	Hot water extraction	RG-I pectic-like polysaccharide, 85 kDa	In vivo	Reduced inflammation, fibrosis and oxidative stress on nonalcoholic steatohepatitis mice	[87]
*Plumbago zeylanica* L.	Hot water extraction	Two low-acetylated RG-I, one with 285 kDa and another with 12 kDa	In vitro	Improved inflammatory damage of LPS+IFN-y induced THP-1 cells through lowering protein levels of CD10, TLR4, and MyD88	[88]
*Auricularia auricula*	Hot water extraction	Polysaccharide composed of Gal, Fru, Ara, Man, Rha and Glc, 140 kDa	In vitro	Mitigation of cell fibrosis and glycosylation through RAGE/TGF-b/NOX4	[89]
*Ribes odoratum* Wendl.	Hot water extraction	One polysaccharide with two peaks, with 8 and 4 kDa	In vivo	Reduced high-fat-diet-associated liver damage and inflammatory markers	[90]

## Data Availability

Data will be made available upon request.
